# Complications in cranioplasty after decompressive craniectomy: timing of the intervention

**DOI:** 10.1007/s00415-020-09695-6

**Published:** 2020-01-17

**Authors:** Taco Goedemans, Dagmar Verbaan, Olivier van der Veer, Maarten Bot, René Post, Jantien Hoogmoed, Michiel B. Lequin, Dennis R. Buis, W. Peter Vandertop, Bert A. Coert, Pepijn van den Munckhof

**Affiliations:** 1 Amsterdam Medical Center, Neurosurgical Center Amsterdam, Amsterdam University Medical Centers (UMC), University of Amsterdam, Meibergdreef 9, Room H2-241, Meibergdreef 9, 1105 AZ Amsterdam, The Netherlands; 2 Department of Neurosurgery, Medical Spectrum Twente, Koningsplein 1, 7512 KZ Enschede, The Netherlands

**Keywords:** Cranioplasty, Decompressive craniectomy, Hydrocephalus, Postoperative complications, Timing

## Abstract

**Objective:**

To prevent complications following decompressive craniectomy (DC), such as sinking skin flap syndrome, studies suggested early cranioplasty (CP). However, several groups reported higher complication rates in early CP. We studied the clinical characteristics associated with complications in patients undergoing CP, with special emphasis on timing.

**Methods:**

A single-center observational cohort study was performed, including all patients undergoing CP from 2006 to 2018, to identify predictors of complications.

**Results:**

145 patients underwent CP: complications occurred in 33 (23%): 18 (12%) epi/subdural hemorrhage, 10 (7%) bone flap infection, 4 (3%) hygroma requiring drainage, and 1 (1%) post-CP hydrocephalus. On univariate analysis, acute subdural hematoma as etiology of DC, symptomatic cerebrospinal fluid (CSF) flow disturbance (hydrocephalus) prior to CP, and CP within three months after DC were associated with higher complication rates. On multivariate analysis, only acute subdural hematoma as etiology of DC (OR 7.5; 95% CI 1.9–29.5) and symptomatic CSF flow disturbance prior to CP (OR 2.9; 95% CI 1.1–7.9) were associated with higher complication rates. CP performed within three months after DC was not (OR 1.4; 95% CI 0.5–3.9). Pre-CP symptomatic CSF flow disturbance was the only variable associated with the occurrence of epi/subdural hemorrhage. (OR 3.8; 95% CI 1.6–9.0)

**Conclusion:**

Cranioplasty has high complication rates, 23% in our cohort. Contrary to recent systematic reviews, early CP was associated with more complications (41%), explained by the higher incidence of pre-CP CSF flow disturbance and acute subdural hematoma as etiology of DC. CP in such patients should therefore be performed with highest caution.

## Introduction

Decompressive craniectomy (DC) can be a lifesaving measure in patients with refractory intracranial hypertension due to space-occupying infarct, traumatic brain injury, aneurysmal subarachnoid hemorrhage, and cerebral venous thrombosis [1-4]. When patients survive and the cerebral swelling has subsided, they need to undergo cranioplasty (CP) to restore cerebral protection and craniofacial cosmesis, and to prevent or treat post-craniectomy complications such as sinking skin flap syndrome [5, 6].

Malcolm et al. recently reported that early CP is associated with greater neurological improvement, and therefore advocate early CP [7]. Although CP is not a complex surgical procedure, high complication rates up to 37% have been published, without an encompassing theory as to causes or risk factors [8]. Meta-analyses by Xu et al. and Malcom et al. showed comparable complication rates between early CP (within three months after DC) and late CP (more than three months after DC) [9, 10]. However, several studies included in the meta-analysis of Malcolm et al. and studies published here after, did report significantly higher complication rates in patients undergoing early CP [11-15].

To further investigate the remarkably high complication rate in patients undergoing CP, we analyzed the long-term complication rate in a large cohort of consecutive patients undergoing CP after DC, and analyzed patient characteristics for predictors of complications, with a special emphasis on timing of the procedure.

## Methods

### Patient population

This retrospective case series included all patients who underwent CP after DC in our university medical center between October 1, 2006 and October 1, 2018. Indications for DC included space-occupying hemispheric brain infarct, aneurysmal subarachnoid hemorrhage, traumatic brain injury, intracerebral hemorrhage, cerebral venous thrombosis, infection, and brain tumor. Patients were selected from a retrospectively created operative database that included all patients undergoing DC in the same time frame [3, 16, 17]. Because this was an observational study, formal approval was waived by the institutional ethical review board of our hospital and patient consent was not required. The STROBE guidelines were used to report this observational study accurately and completely [18].

### Cranioplasty

CP was performed during the initial hospitalization for DC or in an elective setting, when there were no signs of persistent brain swelling [19]. In elective patients, CP was usually performed in the weeks/months following the first follow-up visit to the neurosurgical outpatient clinic (scheduled eight weeks after discharge from the hospital, while the patient was in a rehabilitation center/nursing home). In the interim, patients wore a custom-fitted protective plaster helmet. CT-imaging was performed, although not routinely, for control of intracranial structures or for three-dimensional reconstruction of the skull defect for prefabrication of patient specific implant molds. Before the year 2016, autologous bone flap was used when available, after having been cryopreserved at − 80 °C in the hospital immediately after DC. Since the year 2016, we routinely perform alloplastic CP using polymethylmethacrylate in a customized three-dimensional mold, to avoid resorption [20, 21]. In patients with symptomatic disturbances in CSF flow, the attending neurosurgeon decided whether and when an in situ ventricular- or lumbar-peritoneal shunt or external ventricular/lumbar shunt would be preoperatively closed. Intraoperatively, the attending neurosurgeon decided whether CSF drainage by lumbar/ventricular puncture was needed to replace the bone flap without increasing the intracranial pressure, how many central tack-up sutures were used, how the graft was fixed [non-absorbable sutures, titanium osteoplastic miniplates and screws, or titanium Craniofix (Aesculap AG, Tuttlingen, Germany)], and whether an epidural or subgaleal drain was left behind.

### Data collection

Data sources included hospital records from our own institution, and the referring hospital when applicable, rehabilitation summaries, and correspondence of neurologists, nursing home physicians and general practitioners caring for the patients before and after discharge from our institution. These records were analyzed for demographic characteristics, etiology of brain injury, use of anticoagulation or antiplatelets, presence of comorbidities, symptomatic disturbances in CSF flow, and timing of CP. Considering timing of CP, patients were dichotomized by ≤ 3 months or > 3 months after DC, similar to recent systematic reviews [9, 10].

Piedra et al. and Tsang et al. found that the presence of a permanent pre-CP CSF shunt was significantly associated with complications [22, 23]. Tsang et al. suggested that external lumbar/ventricular CSF drainage prior to CP would be a better alternative in patients who are CSF drainage-dependent at the time of CP. To elucidate the supposedly higher complication rate in patients receiving CSF shunting/drainage, patients were scored as suffering from “symptomatic disturbance in CSF flow prior to CP” when they were treated for symptomatic, CT-scan confirmed hydrocephalus prior to CP. These patients were subdivided into the following categories: patients with either (1) a permanent ventricular-peritoneal (VP) shunt or lumbar-peritoneal shunt, or (2) an external ventricular or lumbar shunt, or (3) patients without a shunt in situ but receiving preoperative pressure-relieving lumbar CSF punctures. Notably, patients without symptomatic disturbance in CSF flow prior to CP but who required intraoperative CSF drainage by lumbar/ventricular puncture to replace the bone flap without increasing the intracranial pressure, were not classified as suffering from “symptomatic disturbance in CSF flow prior to CP”.

CT scans were evaluated by two reviewers (TG and PvdM) to determine the nature of the underlying pathology, the extent of midline shift, and any associated intracranial injuries. If applicable, CT scans were used to calculate the brain sunken ratio, i.e., the ratio of the median length from scalp to midline to the length from midline to contralateral inner table of the skull at the CT-section of maximum size of the craniectomy [24]. Outpatient CT scans, or CT scans within seven days prior to CP without signs of intracranial hypertension were considered applicable for the calculation of the brain sunken ratio.

The following complications occurring within 12 months after CP were noted: 1. symptomatic hemorrhage, 2. bone flap infection, 3. symptomatic hygroma, and 4. symptomatic hydrocephalus requiring implantation of a VP shunt in patients without symptomatic disturbance in CSF flow prior to CP. Foreseen implantation of a VP shunt in patients with pre-CP symptomatic disturbance in CSF flow was not considered as a post-CP complication.

With the collected data, predictors of overall complications were identified. Since different complications can have different risk factors, we also identified predictors of the most frequently occurring complications (i.e., postoperative hemorrhage and postoperative infection) separately.

### Data analysis

Continuous variables were tested for normal distribution using the Shapiro–Wilk test. A variable was considered normally distributed if the Shapiro–Wilk test was > 0.9, otherwise the variable was considered as not normally distributed. Means (± standard deviation, SD) are given for continuous variables with a normal distribution and medians (interquartile range, IQR, 25–75%) are given for not normally distributed continuous variables. To compare demographic and baseline characteristics between patients with versus patients without complications after CP, univariate statistical analysis was performed. The 2-tailed *t* test (for comparisons of normally distributed continuous variables), Mann–Whitney *U* test (for comparisons of continuous variables without a normal distribution), Fisher’s exact test (for analysis of 2 × 2 tables), and chi-square test (for analysis of *N* × 2 contingency tables) were done when appropriate to identify differences between groups. Results with a *p* < 0.05 were considered statistically significant. Additionally, multivariate logistic regression analysis was performed including significant associations of complications on univariate analysis. IBM SPSS Statistics 24.0 (IBM Corporation) was used for calculations.

## Results

### Patient characteristics

A total of 310 consecutive patients underwent supratentorial DC between October 1, 2006 and October 1, 2018. One hundred and thirty-two (43%) patients died, 9 (3%) did not undergo CP, and 24 (7%) were lost to follow-up. The remaining 145 (47%) patients, with a mean ± SD age of 44.3 ± 14.7 years, underwent CP after a median delay of 136 (IQR 91–210) days after DC. Diagnosis for which DC was performed in these 145 patients were space-occupying hemispheric brain infarct (35%), aneurysmal subarachnoid hemorrhage (20%), traumatic brain injury (19%), intracerebral hemorrhage (15%), cerebral venous thrombosis (9%), infection (1%), and brain tumor (1%). Thirty-three (23%) patients developed a post-CP complication during a median follow-up of 12 months. Patient characteristics for the CP cohort in total, and sorted by presence or absence of complications, are presented in Table 1.

**Table 1:** Baseline characteristics of 145 patients undergoing cranioplasty after decompressive craniectomy

Characteristics	Total (*n* = 145)	Without complication (*n* = 112)	With complication (*n* = 33)	*p* value^a^
Gender, female, *n* (%)	78 (54)	60 (54)	18 (55)	
Age, mean ± SD	44.3 ± 14.7	43.4 ± 14.8	47.3 ± 14.0	
History				
Hypertension, *n* (%)	29 (20)	23 (21)	6 (18)	
Diabetes mellitus, *n* (%)	6 (4)	6 (5)	0	
Ischemic heart disease, *n* (%)	7 (5)	6 (5)	1 (3)	
Antiplatelet agent, *n* (%)	15 (10)	12 (11)	3 (9)	
Gore-tex® skin plasty during DC	5 (3)	3 (3)	2 (6)	
Surgery between DC and cranioplasty, *n* (%)	37 (25)	25 (22)	12 (36)	
Etiology of DC				0.012
Infarction, *n* (%)	51 (35)	46 (41)	5 (15)	0.009
SAH, *n* (%)	29 (20)	20 (18)	9 (27)	
TBI:				
aSDH, *n* (%)	13 (9)	4 (4)	9 (27)	< 0.001
aEDH, *n* (%)	6 (4)	5 (4)	1 (3)	
Contusion, *n* (%)	6 (4)	4 (4)	2 (6)	
Gunshot, *n* (%)	2 (1)	2 (2)	0	
ICH, *n* (%)	21 (15)	16 (14)	5 (15)	
CVT, *n* (%)	13 (9)	12 (11)	1 (3)	
Infection, *n* (%)	2 (1)	2 (2)	0	
Tumor, *n* (%)	2 (1)	1 (1)	1 (3)	
Left side of cranioplasty, *n* (%)	66 (45)	50 (45)	16 (49)	
Bifrontal cranioplasty, *n* (%)	4 (3)	3 (3)	1 (3)	
Pre-CP symptomatic CSF disturbance, *n* (%)	32 (22)	18 (16)	14 (42)	0.002
Pre-CP VP shunt, *n* (%)	6 (4)	4 (4)	2 (6)	
Not closed	4	4	0	
Programmable valve at highest resistance	2	0	2	
Pre-CP not closed LP shunt, *n* (%)	2 (1)	1 (1)	1 (3)	
Pre-CP closed EL-shunt	3 (1)	3 (3)	0	
Pre-CP closed EV-shunt, *n* (%)	9 (6)	4 (4)	5 (15)	
No drain in situ pre-CP^b^	12 (8)	6 (5)	6 (18)	
DC-CP interval				
Median days (IQR)	136 (91–210)	141 (103–217)	101 (47–168)	0.008
≤ 3 months, *n* (%)	37 (26)	22 (20)	15 (46)	0.004
Residence pre-CP				
During initial hospitalization for DC, *n* (%)	31 (21)	17 (15)	14 (42)	0.001
At a RC, *n* (%)	52 (36)	46 (41)	6 (18)	
At a nursing home, *n* (%)	15 (10)	10 (9)	5 (15)	
At home, *n* (%)	47 (32)	39 (35)	8 (24)	
Pre-CP CT finding in 75 patients				
Midline-shift, mm, median (IQR)	3 (2–6)	3 (2–6)	3 (2–8)	
Sunken ratio, mean ± SD^c^	1.05 ± 0.20	1.07 ± 0.18	0.99 ± 0.26	
Surgical expertise				
Resident + attending, *n* (%)	114 (79)	87 (78)	27 (82)	
Attending, *n* (%)	31 (21)	25 (22)	6 (18)	
CP management (*n* = 137^d^)				
Intraoperative lumbar/ventricular drainage^e^	38 (28)	26 (25)	12 (38)	
< 3 central tack-up sutures	105 (77)	80 (76)	25 (78)	
Graft fixation				
Non-absorbable sutures	34 (25)	26 (25)	8 (25)	
Titanium osteoplastic plates/screws	67 (49)	48 (46)	19 (59)	
Titanium Craniofix (Aesculap AG)	36 (26)	31 (30)	5 (16)	
Epidural/subgaleal drain^f^	40 (29)	32 (31)	8 (25)	
Using artificial bone, *n* (%)	33 (23)	23 (21)	10 (30)	
Hospital stay post-CP, days, median (IQR)	3 (2–8)	3 (2–4)	15 (6–32)	< 0.001

*aEDH* acute epidural hematoma, *aSDH* acute subdural hemorrhage, *CP* cranioplasty, *CSF* cerebrospinal fluid flow, *CVT* cerebral venous thrombosis, *DC* decompressive craniectomy, *EV* external ventricular, *ICH* intracerebral hematoma, *IQR* interquartile range, *LP* lumbar-peritoneal shunt, *N* number of patients, *RC* rehabilitation center, *SD* standard deviation, *TBI* traumatic brain injury, *VP* ventricular-peritoneal

^a^2-tailed *t* test for means, Mann–Whitney *U* test for medians, Fisher’s exact test for binary variables, and chi-square test for ordinal variable (pupillary light reflexes pre-DC), only *p* < 0.05 shown

^b^CSF disorder, without shunt in situ: patients with hydrocephalus, receiving pressure-relieving CSF taps pre-cranioplasty

^c^Sunken ratio: the ratio of A (the median length from scalp to midline) to B (the length from midline to inner table skull at this level) at the CT-section of maximum size craniectomy

^d^In eight patients no complete operational record was available

^e^Twenty-two of 38 patients receiving intraoperative drainage had no clinically symptomatic disturbances in cerebrospinal fluid

^f^Three patients with epidural drain, 37 with subgaleal drain

### Predictors of complications

Eighteen (12%) patients suffered from symptomatic postoperative hemorrhage (epidural hematoma in 17, and subdural hematoma in one), requiring surgical evacuation in 17. One patient, with a pre-CP GCS score of 10 (E4M4V2) and a restriction of treatment (“do not resuscitate”, “no artificial respiration”), died one day post-CP due to a massive postoperative epidural hemorrhage. In all 10 (7%) patients with postoperative infection, the bone flap was removed. A newly made alloplastic CP was placed in the following months. Four (3%) patients with postoperative hygroma received burr hole drainage. One (1%) out of 113 patients without pre-CP symptomatic disturbance in CSF flow suffered from post-CP symptomatic hydrocephalus and received a VP shunt (Table 2). Thirteen (50%) out of the 26 patients with pre-CP external CSF drainage required post-CP VP shunt implantation (as stated in the Methods, these foreseen VP shunt implantations were not noted as complication). In the remaining 13 (50%), CP resolved the symptomatic hydrocephalus. Eleven (79%) of the 14 post-CP VP shunts were implanted in patients undergoing CP within three months after DC.

**Table 2:** Postoperative complications in 33 of 145 patients undergoing cranioplasty

Complication	Number of patients (% of total)
Postoperative hemorrhage	18 (12)
Symptomatic EDH	17
Evacuation of EDH	16
Died due to EDH	1
Symptomatic SDH, with evacuation	1
Postoperative infection, with removal of the graft	10 (7)
Hygroma requiring burr hole drainage	4 (3)
Hydrocephalus requiring VP shunt placement	1 (1)

*EDH* epidural hematoma, *SDH* sundural hematoma, *VP shunt* ventriculoperitoneal shunt

**Fig. 1:**
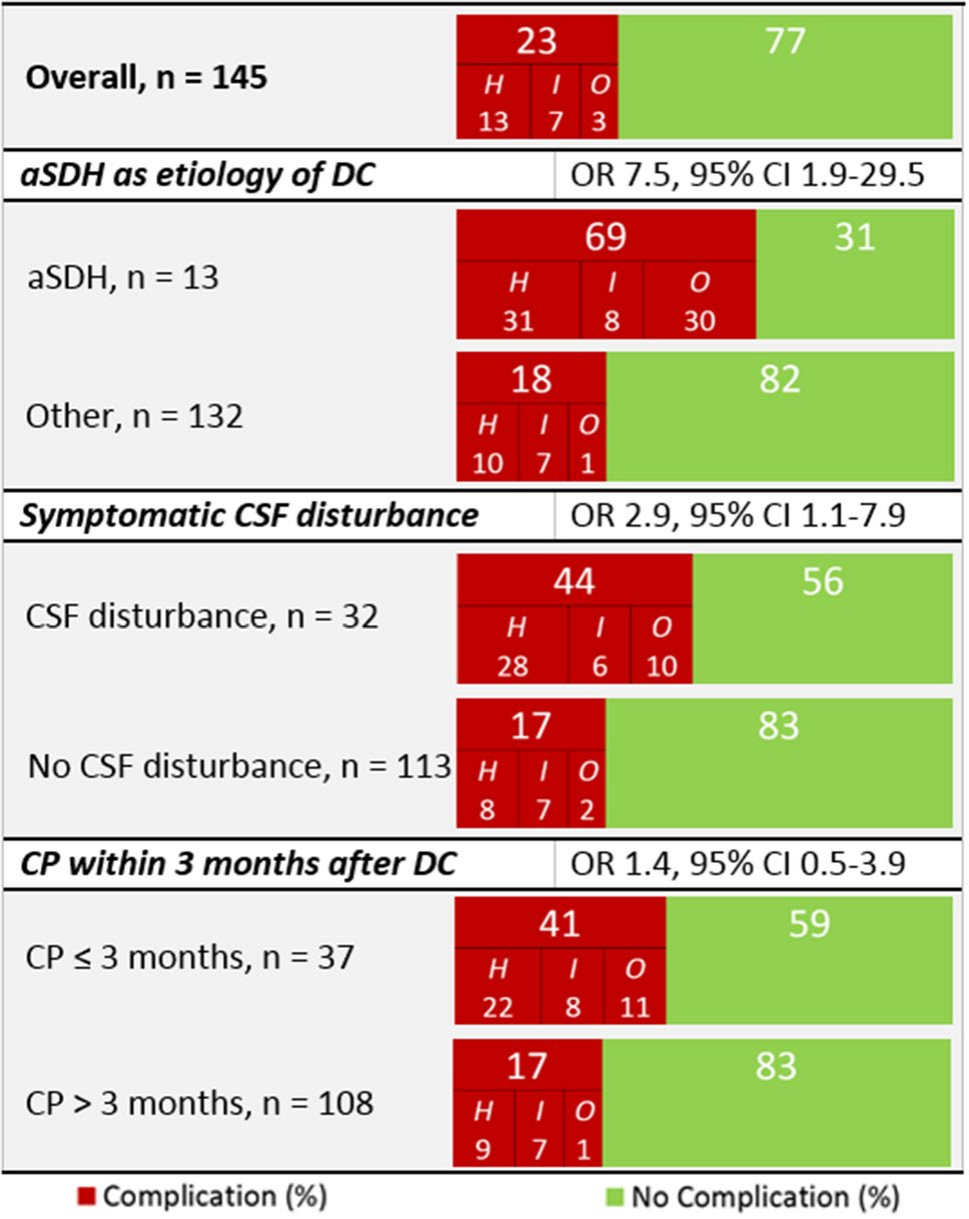
Multivariate analysis of associations with high complication rates in 145 patients undergoing cranioplasty (CP); *aSDH* acute subdural hematoma, *CI* confidence interval, *CSF* cerebrospinal fluid flow, *DC* decompressive craniectomy, *H* postoperative hemorrhage, *I* infection, *O* other complications, *OR* odds ratio, *n* number of patients

Acute subdural hematoma (aSDH) after high-energy trauma as etiology of DC (OR 10.1; 95% CI 2.9–35.6), pre-CP symptomatic disturbances in CSF flow (OR 3.8; 95% CI 1.6–9.0), and CP within three months after DC (OR 3.4; 95% CI 1.5–7.8) were associated with higher complication rates on univariate analysis. On multivariate analysis, aSDH as etiology of DC and pre-CP symptomatic CSF flow disturbance remained associated with higher complication rates, whereas CP performed within three months after DC was not (OR 1.4; 95% CI 0.5–3.9) (Fig. 1). Pre-CP symptomatic disturbance in CSF flow was the only variable associated with the occurrence of post-CP symptomatic epi/subdural hemorrhage (OR 3.8; 95% CI 1.6–9.0).

### Timing of cranioplasty

**Fig. 2:**
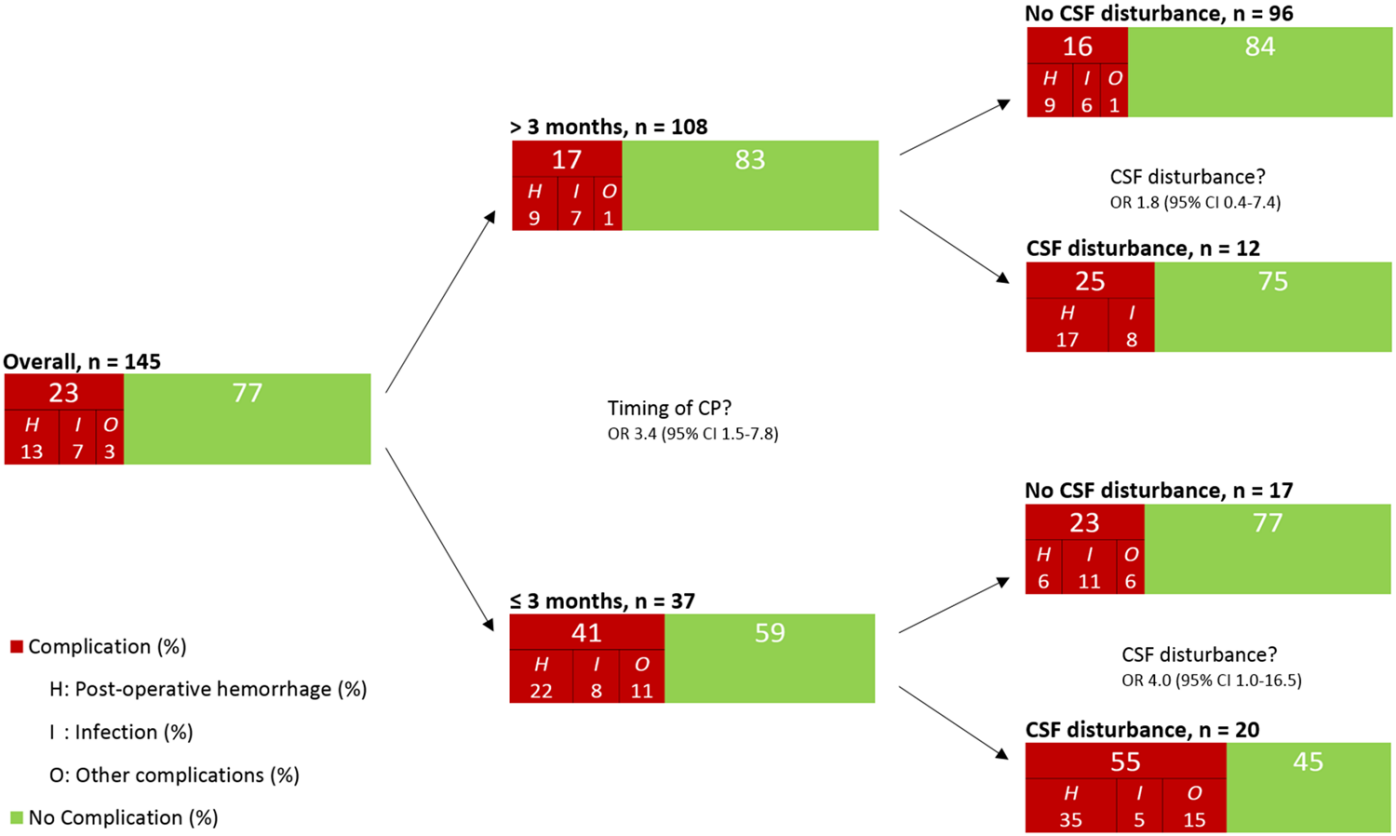
Complication rate of 145 patients undergoing cranioplasty (CP), stratified for timing of CP, and preoperative symptomatic disturbances in cerebrospinal fluid flow; *CI* confidence interval, *CSF* cerebrospinal fluid flow, *OR* odds ratio, *n* number of patients

Considering the impact of timing of CP on postoperative complications, aSDH as etiology of DC (*p* < 0.0001), and pre-CP symptomatic disturbance in CSF flow (*p* < 0.0001) were more frequently present for patients undergoing CP within three months after DC. (Table 3) Twenty-nine out of 37 patients who received CP within three months were still hospitalized after the initial DC, 15 (52%) of these suffered a complication. In contrast, only one (13%) of the eight outpatients who received CP within three months suffered a complication (*p* = 0.075). The incidence of complications in patients receiving CP within three months after DC differed when dichotomizing by “pre-CP symptomatic disturbance in CSF flow” (OR 4.0; 95% CI 1.0–16.5) (Fig. 2).

**Table 3:** Characteristics of 145 patients undergoing cranioplasty categorized by timing within three months after decompressive craniectomy

Characteristics	CP ≤ 3 months *n* = 37	CP > 3 months *n* = 108	*p* value^a^
Gender, female, *n* (%)	24 (65)	54 (50)	
Age, mean ± SD	43.4 ± 16.3	44.6 ± 14.1	
Pre-CP CSF disturbance, *n* (%)	20 (54)	12 (11)	< 0.0001
aSDH as etiology of DC, *n* (%)	10 (27)	3 (3)	< 0.0001
CP during initial hospitalization for DC, *n* (%)	29 (78)	2 (2)	< 0.0001
Complication, *n* (%)	15 (41)	18 (17)	0.004
Postoperative hemorrhage, *n* (%)	8 (22)	10 (9)	
Postoperative infection, *n* (%)	3 (8)	7 (6)	

*aSDH* acute subdural hematoma, *CP* cranioplasty, *CSF* cerebrospinal fluid flow, *DC* decompressive craniectomy, *n* number of patients, *SD*standard deviation

^a^2-tailed *t* test for means (age), and Fisher’s exact test for binary variables, only *p* < 0.05 shown

### Brain sunken ratio

Seventy patients did not undergo CT-imaging within seven days before CP, or at the outpatient clinic. There was no difference in complication rate between patients who did receive a pre-CP CT-scan and those who did not (27% versus 23%, respectively; *p* = 0.71). The brain sunken ratio was calculated for 75 patients: 23 patients underwent CT-imaging in a clinical setting within seven days before CP with a median delay between the CT scan and CP of two (IQR 1–5) days, 52 patients underwent an outpatient CT scan with a median delay between the CT scan and CP of 49 (IQR 3–88) days. No difference in brain sunken ratio was found, when patients were dichotomized by occurrence of postoperative complications: 1.07 ± 0.18 in patients without complications versus 0.99 ± 0.26 in patients with complications (p = 0.250).

### Bone flap resorption

In 112 (76%) patients, autologous bone grafts were used for cranioplasty. There was no difference in complication rate between allograft and autologous graft during the first post-CP months. (Table 1) However, in 18 (16%) of the 112 patients with autologous grafts, bone flap resorption occurred after 28 ± 19 months. In 12 of these 18 patients, a second CP (with allograft) was needed. One of them suffered a postoperative epidural hemorrhage requiring evacuation and craniectomy, followed by tertiary CP after seven months.

## Discussion

We reviewed the long-term complication rate of patients undergoing cranioplasty (CP) following decompressive craniectomy (DC) by analyzing a 13-year, single-center, consecutive patient cohort. CP-related complications occurred in 23%, similar to many published studies of comparably sized cohorts [25-30]. The presence of symptomatic disturbance in CSF flow prior to CP and acute subdural hematoma as etiology of DC were associated with higher complication rates. Pre-CP symptomatic CSF flow disturbance was the only associated risk factor for the occurrence of post-CP symptomatic epi/subdural hemorrhage*.*

### Timing of cranioplasty

Systematic reviews by Xu et al. and Malcolm et al. showed comparable complication rates between early CP (within three months after DC) and late CP (more than three months after DC) [9, 10]. In our study*,* early CP was associated with more complications, but on multivariate analysis this higher complication rate was explained by the higher incidence of pre-CP CSF flow disturbance and acute subdural hematoma as etiology of DC among early CP patients. Patients with CSF flow disturbance were especially at risk for postoperative hemorrhage. Xu et al., Malcolm et al., and also the more recently published large cohort study by Morton et al. reported a higher rate of postoperative hydrocephalus requiring a shunt in patients undergoing CP within three months [9, 10, 29]. However, they did not report data on the presence of pre-CP hydrocephalus, which may have been more present among early CP patients. In our cohort, the percentage of patients requiring a post-CP shunt was higher in the early CP group compared to the late CP group (30% versus 3%), but this was explained by the higher incidence of pre-CP CSF flow disturbance in the early CP group. We did not consider (foreseen) VP shunt implantation in patients suffering from pre-CP hydrocephalus as a complication.

### Why is CSF flow disturbance prior to CP associated with more complications?

Similar to our findings, Piedra et al. and Tsang et al. found that the presence of a permanent pre-CP CSF shunt was significantly associated with complications [22, 23]. Tsang et al. suggested that external lumbar/ventricular CSF drainage prior to CP would be a better alternative in patients who are CSF drainage-dependent at the time of CP. Our results do not support this suggestion: five of the 12 patients with an external lumbar/ventricular CSF drain prior to CP developed a complication. Hypothetically, patients treated for pre-CP CSF flow disturbance are at higher risk of complications due to potential pre-CP overdrainage of CSF, causing relatively negative intracranial pressure which may cause postsurgical hemorrhage. On the other hand, CP may serve to restore normal CSF flow, and sometimes even improve neurologic outcome, especially in patients with clinical signs of sinking skin flap syndrome [5, 8, 11, 31, 32]. Indeed, CP resolved symptomatic hydrocephalus in half of our patients suffering from pre-CP symptomatic hydrocephalus. CSF flow disturbance should therefore not be a reason to restrict performing CP, but the amount of pre-CP CSF drainage should be carefully monitored and, if possible, minimized. Alternatively, a longer interval between DC and CP could be considered to evaluate whether spontaneous recovery of pre-CP hydrocephalus occurs. However this may lead to a longer admission to the hospital or delayed initiation of rehabilitation. In brief, we do not have an easy solution for the remarkably high complication rate in patients with pre-CP hydrocephalus. CP in such patients should be performed with highest caution.

### Brain sunken ratio

We did not find a correlation between the brain sunken ratio and complication rate, contrary to the study by Lee et al. [24]. Unfortunately, we were not able to analyze this brain sunken ratio in all patients: most of our neurosurgeons decided to schedule a patient for CP based on physical examination of the skin flap only, and many patients did not routinely receive pre-CP CT-imaging in a clinically stable setting or at the outpatient clinic. Whether patients received pre-CP CT-imaging was not associated with a difference in complication rate. Additionally, there was a large heterogeneity in time between CT-imaging and CP in the remaining patients who did receive pre-CP CT-imaging. Possibly, future studies can elucidate the possible benefits of routine pre-CP CT-imaging, with added emphasis on timing before CP. Such imaging may also be helpful in patients suffering from pre-CP symptomatic hydrocephalus, treated with external ventricular/lumbar CSF drainage, to carefully monitor the consequences of drainage on brain sinking.

### Limitations of the study and future perspectives

The present study has several limitations. First, data collection was performed retrospectively. Second, CP patients were dichotomized by ≤ 3 months or > 3 months after DC, similar to recent systematic reviews [9, 10]. In our cohort this led to a skewed distribution of timing with only 37 out of the 145 patients falling within the early group. But since 37 is still a substantial number of patients, we consider our conclusions as relatively solid. Third, there was no standardized preoperative treatment protocol in patients with symptomatic CSF flow disturbance, or standardized operative technique of CP. The high variability in pre- and intraoperative treatment was not a prognostic factor of complications, but a more stringent and uniform treatment/surgical protocol may result in less complications [33, 34] For example, to prevent pre-CP overdrainage of CSF, one may close external lumbar/ventricular CSF drains or lumbar/ventricular-peritoneal shunts a minimum number of days before CP. Furthermore, a uniform intraoperative protocol (including a minimum number of tack-up sutures and method of flap fixation) could be implemented. Also, if all patients would receive routine pre-CP CT scans, a potential correlation between the brain sunken ratio and complications rate could be better identified, and could then serve to implement a minimum brain sunken ratio in the treatment protocol. The very high complication rate in the subgroup of patients with symptomatic CSF flow disturbance prior to CP is clearly unacceptably high. We should undertake any effort possible to lower this rate in the coming years.

## Conclusion

Cranioplasty has high complication rates, 23% in our cohort. Contrary to recent systematic reviews showing comparable complication rates between early CP (within three months after DC) and late CP (more than three months after DC), early CP in our cohort was associated with more complications (41%). On multivariate analysis, this higher complication rate was explained by the higher incidence of pre-CP CSF flow disturbance and acute subdural hematoma as etiology of DC among early CP patients. Pre-CP symptomatic CSF flow disturbance was the only associated risk factor for the occurrence of post-CP symptomatic epi/subdural hemorrhage. CP in such patients should therefore be performed with highest caution.
